# Being a good egg in the 21st century

**DOI:** 10.1093/bmb/ldy023

**Published:** 2018-07-31

**Authors:** Richard A Anderson, Evelyn E Telfer

**Affiliations:** MRC Centre for Reproductive Health, Queen’s Medical Research Institute, University of Edinburgh and Institute of Cell Biology, School of Biological Sciences, University of Edinburgh, Edinburgh, UK

**Keywords:** oocyte quality, reproductive ageing, elective oocyte banking, artificial gametes

## Abstract

**Introduction:**

Women are increasingly having children at a later age, but this can conflict with declining fertility in the later 30′s and thereafter.

**Areas of agreement:**

Declining egg quality and quantity with age are well-established, although egg quality can only be surmised from reproductive success or failure.

**Areas of controversy:**

Whether increasing the number of eggs that can be obtained from ovarian stimulation is of value, and whether there are precursor cells within the adult ovary that could become mature eggs.

**Growing points:**

There is increasing use of donated eggs by older women to enhance their chances of conception. The storage of frozen eggs for potential use later in life is also becoming more common.

**Areas timely for developing research:**

Understanding of growth initiation of follicles and development of an artificial ovary may lead to the ability to affect fertility and reproductive lifespan.

## Introduction

Recognition of the fundamental importance of the egg, or more accurately the oocyte, to reproduction is encapsulated in the phrase ‘ex ovo omnia’, represented on the frontispiece of William Harvey’s ‘Exercitationes de Generatione Animalium’ (1651). The central, indeed limiting, role of the oocyte is of ever growing importance in the face of changes both in society and with developments in medical technology. The societal changes are most importantly the ongoing trend for later child birth in developed societies, and as a direct result of the growing number of women in their later reproductive years wishing to start or complete their families. This has resulted in a growing reliance on medical technology to provide solutions to address the underlying decline in female fertility with age. This gives an added dimension to the dramatic growth in recent years in commercial reproductive medicine services in the UK, Europe and worldwide. In the UK this increase is, at least in part, driven by the very limited provision of assisted reproduction by the National Health Service.

**Fig. 1:**
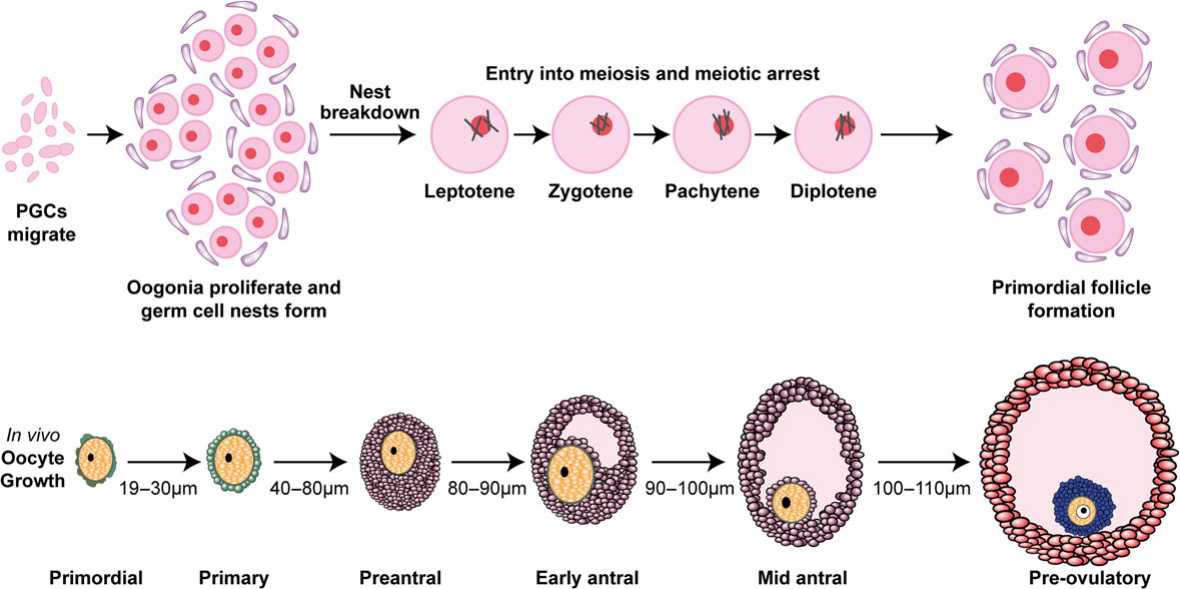
Illustration of primordial follicle development and subsequent growth through preantral and antral stages to the point of ovulation. Primordial germ cells (PGCs) proliferate forming nests, and then enter meiosis. Nest break down forming primordial follicles, which are the non-growing state. A number of these start to grow each day, progressing through the stated phases during which they become gonadotrophin sensitive and then dependent. In the final preovulatory phase, the ovulation-inducing LH surge causes the oocyte to reenter meiosis and be released from the follicle. The sizes given are of the oocyte, not the follicle, showing how that also grows dramatically during this process.

The key biological process underpinning this field is that in humans, ovarian development occurs during fetal life, with the basis for a woman’s fertility and reproductive lifespan laid down before she is born. In the early weeks of development the ovary contains proliferating primordial germ cells (PGCs) but their number is constrained by commitment to meiotic division, which starts in the fourth month of pregnancy.^1^ This change from germ cell division by mitosis to meiosis, which precludes further proliferation, limits the total number of oocytes that can be formed. Following this step, the clusters in which the early oocytes have been developing break down, separating into individual oocytes which associate with surrounding somatic cells to form the pool of primordial follicles (Fig. 1). This is accompanied by extensive germ cell death, with the majority of oocytes being lost. Primordial follicles are the fundamental developmental unit of the human ovary and consist of a single oocyte which is now arrested in early meiosis, surrounded by a single layer of flattened somatic cells and constitute what is known as the ‘ovarian reserve’, the non-growing pool of follicles from which all follicle growth and thus both ovarian endocrine activity and fertility derive. Development from this pool is a continuous process and activation of a small proportion of primordial follicles occurs as soon as follicles are formed thus leading to a depletion of the reserve with time.^2^

While all follicles that start to grow during childhood are destined to degenerate, the activation of the neuroendocrine axis at puberty will provide the support for further growth and hormone production of a proportion of these follicles. This is under stimulatory control from gonadotropin hormone releasing hormone from the hypothalamus of the brain, which results in increased secretion of the hormones luteinising hormone and follicle stimulating hormone from the pituitary. While the early stages of follicle development are primarily under local paracrine control from growth factors produced by the somatic cells in the local environment and other nearby growing follicles,^3^ later stages are increasingly sensitive to and then dependent on LH and FSH secretion, whose subtle variation across the menstrual cycle results in the selection of just one follicle per month for ovulation.^4^ Throughout life, the great majority of follicles become atretic at some point during their development, with 1 in perhaps 300 that start to grow per month resulting in ovulation and thus the potential for pregnancy in a women of reproductive age. Ultimately, menopause occurs when the number of follicles remaining in the ovary is too small to support sufficiently the process of selection for dominance and ovulation each month. This results in the end of the potential for fertility, and a major fall in estrogen production, as that is determined by the presence of large growing follicles, inducing the vasomotor and other symptoms of the menopause.

For as long as a woman continues to ovulate, there is the potential for conception each month but the chance of this declines substantially with age. This is accompanied by an increase in the chance of miscarriage of early pregnancies, with both these factors reflecting declining egg quality.^5^ This age-related decline is attributed to the onset of meiosis during fetal life, discussed below, with the consequent need to maintain chromosomal integrity for decades thereafter. In the face of these factors, there have been substantial changes in the age distribution of women giving birth in recent decades, with recent the UK data showing steady increases in the number of births from women in their thirties and indeed, forties over the last 30 years with equivalent decreases in women in their teens and early twenties.^6^ Indeed, in 2015 the birth rate in women aged 40 and over was greater than that for women under the age of 20. While there is much to celebrate in the falling teenage pregnancy rate, there seems little likelihood of any change in the increasing wish of women in their later thirties and early forties to have a baby, and indeed to start rather than complete their family at those ages. While there are many important social and cultural aspects to this, we will consider only what medical technology may be able to contribute.

The importance of entry into meiosis preventing further germ cell proliferation during fetal life has been mentioned above. While therapeutic intervention in this process seems impossible at this point, it is also relevant that there is growing evidence of the importance of the intrauterine environment to subsequent adult health, and it seems highly likely that this will apply to reproductive function as well as other areas. An example of where intrauterine exposures are now established to have a role in reproductive development is the evidence implicating analgesic exposure during pregnancy to abnormal male reproductive function such as testicular maldescent. *In utero* exposure to smoking has also been implicated as a potential factor affecting germ cell development in both males and females. Recent data suggest that analgesic exposure in utero may also affect ovarian germ cell development.^7^ The key molecular processes involved in early meiosis also have implications for adult oocyte quality.^8^ The correct formation of crossovers during meiotic recombination is required to reduce the chance of subsequent chromosomal missegregation. It is essential that sister chromatids are held together until meiosis resumes, which is not until the time of ovulation, thus potentially decades after the onset and then early arrest of the early stages of meiosis. This chromatid cohesion is mediated by cohesin rings. These are established during fetal life during early meiosis, and there is evidence that they cannot subsequently be topped-up or replenished, and this their correct number and function appears necessary for the preservation of egg quality through the reproductive years. Early loss of these cohesin rings is thought to be the main cause of oocyte aneuploidy, which is a key cause of embryo loss and miscarriage.^8^ While much of this data derives from animal models, there is some evidence to support its relevance in human oocytes as well. A reduced number of mitochondria in the oocyte, has also been proposed as a cause of reduced oocyte quality, with therefore the potential for benefit from supplementation. This has been confirmed in a pig model, with improved embryo development following mitochondrial supplementation of oocytes with low, but not normal, numbers of mitochondria.^9^

The next key stage in optimising the number of eggs potentially available would be through increasing the number of primordial follicles that start to grow, by analogy opening the tap on the supply of growing follicles. Understanding of the details of how primordial follicle activation occurs is still limited although some essential biochemical pathways have been clearly demonstrated to be implicated.^10^ It is at least theoretically possible to increase the activity of these pathways thus causing increased follicle activation but in vitro experiments thus far indicate that this is likely to be associated with a disruption in the tightly coordinated development of the oocyte in its surrounding granulosa cells, with potential loss of quality.^11^ More tractable are the later stages of follicle development, and indeed supplementing FSH concentrations to prevent follicle atresia is the basis for the well-established ovarian stimulation protocols used for IVF. There is currently much interest clinically in whether alternative hormonal approaches such as the use of androgens may increase the supply of early growing follicles and thus allow more to progress through to stages at which gonadotrophin stimulation regimens will support further development to mature stages.^12^

An alternative strategy to increase the number of oocytes is to activate and grow large numbers of immature oocytes to maturity *in vitro*.^13^ These techniques would be particularly useful in the context of fertility preservation, which is the storage of oocytes, embryos or specifically relevant here of ovarian tissue in women prior to fertility-damaging treatment for conditions such as cancers.^14^ The preservation and restoration of female fertility following chemotherapy by cryopreservation and transplantation of ovarian tissue has now resulted in over 100 live births worldwide^15^ and whilst the success of this strategy has given tangible hope to patients of conceiving and bearing their own child there remains a risk of reintroduction of the cancer, which means some patients could not be considered for this procedure.

Such patients might also benefit from advances in *in vitro* growth of follicles and maturation systems designed to support follicle development from the early and more advanced stages, to produce mature oocytes capable of fertilisation.^16,17^ A focus of research has been on obtaining *in vitro* grown oocytes either starting from the primordial stage of follicle development within ovarian fragments^16–18^ or from isolated growing follicles (preantral follicles) *in vitro*.^19,20^ A proportion of human oocytes grown from primordial stages are able to resume meiosis and reach Metaphase II (the stage at which fertilisation happens) thus demonstrating meiotic maturity.^17^ Whilst this source of oocytes is promising, there is still a great deal of work required to determine how normal *in vitro* grown oocytes are.

More futuristic sources for obtaining human oocytes are the creation of so called ‘artificial’ or in vitro grown gametes from embryonic stem cells, from induced pluripotent stem cells, or from endogenous cells within the ovary that have the potential to become oocytes, often called oogonial or germline stem cells.^21,22^ Putative human oogonial stem cells have been isolated from adult human ovaries^23,24^ and these cells have some potential to form oocyte/follicle like structures *in vitro*. The concept that women are born with a finite number of eggs has been a cornerstone of reproductive biology for the last 60 years, but has now been charged by the possible existence of these cells within the adult ovary, that may have the potential to form mature oocytes. Since the initial descriptions of oogonial stem cells,^25,26^ there has been substantial controversy as to whether they may or may not indeed exist, and even if they do, what might be their development potential. Only very few groups have successfully isolated these cells, but if confirmed they would have very substantial therapeutic applications in potentially allowing the formation of new oocytes, potentially of high quality as they would not have been subject to the normal long wait between formation and later development. Any significant physiological role as a contributor to post-natal ovarian function, however, seems very unlikely at present. Oocyte development in the mouse can be recapitulated *in vitro* starting from both PGCs or, in experiments even more removed from normal physiology, from induced pluripotent stem cells, which can become immature oocytes under appropriate conditions with those oocytes going on to produce viable and fertile offspring.^27,28^

Obtaining oocytes in this way could alleviate the need for donor eggs and allow women who cannot produce mature oocytes the chance of having their own genetic offspring, although this remains a distant goal at present. The development of these types of techniques would also allow insights into the basic science of oogenesis, folliculogenesis and meiosis as well as providing the potential for new assisted reproductive technologies. These methods remain experimental and the lack of successful clinical translation is due in part to the exquisite sensitivity and complexity of follicle maturation in large mammals. Before clinical application can ever be realised appropriate testing for epigenetic and meiotic normality of *in vitro* grown or derived oocytes will be required.

In recent years, a steady accumulation of work has been published demonstrating an alternative strategy to *in vitro* growth or transplant of potentially cancer-infected tissue by creating an ‘artificial ovary’. The concept of the artificial ovary is of semi-solid three-dimensional scaffold structure capable of supporting the development and maturation of ovarian cells seeded into or onto its surface whilst also supporting endocrine function of the developing follicles. This involves utilising supportive matrices capable of being seeded with isolated ovarian follicles (that may have been individually frozen-thawed), and transplanting these composites into the pelvic cavity. Encapsulating matrices for immature follicle development have been devised from a number of biomaterials including agarose,^29^ alginate,^30^ gelatin^31^, fibrin^32^ and aim to replicate the physiological norms of an endogenous ovary as closely as possible. The majority of this work has been conducted using mouse models but is encouraging with structures capable of supporting the growth and maturation of murine primordial follicles and the birth of live pups to ovariectomised mice after surgical placement of artificial implants. Whilst these bioengineering achievements are remarkable, the length of time necessary for immature follicles to develop is very different to that in higher animals and there are concerns about the ability to ‘upscale’ and translate findings for clinical application.

A more realistic option for the foreseeable future is the development of both the technology and clinical services for freezing eggs at a young age for later use. The development of oocyte vitrification in recent years has dramatically improved the survival of frozen eggs and indeed vitrified oocytes can have the same developmental competence as fresh ones. Vitrification is ‘flash freezing’ involving very rapid temperature changes, which prevent the formation of damaging ice crystals within oocytes. This has led to the development of services for what is often termed ‘social egg freezing’ as well as its use for specific medical indications, such as for women with cancer prior to chemotherapy that will markedly impact their subsequent fertility. Controversially, it has also been offered by some employers.^33^ The slightly pejorative tone of the term ‘social egg freezing’ is resulting in it increasingly being replaced by ‘elective egg banking’. There are rapid commercial developments in this field and clearer data on likely success rates are now becoming available highlighting the impact both of the woman’s age and the number of eggs she is able to store.^34^ In the UK, the duration of storage is limited to 10 years, which is very unhelpful to younger women, in whom of course this approach would be most successful. Ovarian tissue cryopreservation, developed for fertility preservation for girls and young women with cancer as discussed above, would also, at least in theory, offer an alternative approach for women wishing to preserve their fertility for non-medical reasons. It does, however, require a surgical procedure to remove the ovarian tissue as well as its subsequent replacement and thus at the moment it appears to be used only for medical indications.^15^

If the number and quality of one’s own eggs is compromised, then using someone else’s is a possibility. The use of egg donation has clearly shown that egg quality is indeed the key determinant of the decline in success rate in assisted reproduction with age, with the chance of birth being dependent on the age of the egg donor, not the recipient.^35^ Although initially used only for women who had no oocytes of their own egg following cancer treatment or other causes of premature ovarian insufficiency this approach is increasingly used by premenopausal but older women. It is undoubtedly a technically very successful treatment, increasing the cumulative live birth rate for women aged over 40 from under 10% to 30% per embryo transfer.^36^ The limiting factor is often the number of women coming forward as potential egg donors but in many countries IVF clinics have been very successful in establishing a sufficient supply of oocytes for donation, and indeed are increasingly establishing ‘egg banks’ of frozen oocytes so that potential recipients do not have to have their treatment synchronised with that of the donor. The marked differences in availability of this by country have resulted in what is sometimes termed ‘reproductive tourism’, although that term is increasingly replaced by ‘cross border reproductive health care’.

## Conclusions

In summary, therefore, there are very clear and indeed growing issues and pressures surrounding the need to be ‘a good egg’ in the 21st century. This subject is at the interface of both social changes and technological developments in reproductive medicine, with this interaction providing both a drive to progress and public debate. In the UK, there is a very clear regulatory framework which the great majority of those working in this field strongly supports, although its basis in primary legislation does not readily allow for change. It seems clear that the need for public debate will, if anything, increase in coming years as the ability to manipulate human reproductive function increases.
